# Using Information Interaction to Discover Epistatic Effects in Complex Diseases

**DOI:** 10.1371/journal.pone.0076300

**Published:** 2013-10-23

**Authors:** Orlando Anunciação, Susana Vinga, Arlindo L. Oliveira

**Affiliations:** INESC-ID/Instituto Superior Técnico, University of Lisbon, Portugal; Cleveland Clinic Lerner Research Institute, United States of America

## Abstract

It is widely agreed that complex diseases are typically caused by the joint effects of multiple instead of a single genetic variation. These genetic variations may show stronger effects when considered together than when considered individually, a phenomenon known as epistasis or multilocus interaction. In this work, we explore the applicability of information interaction to discover pairwise epistatic effects related with complex diseases. We start by showing that traditional approaches such as classification methods or greedy feature selection methods (such as the Fleuret method) do not perform well on this problem. We then compare our information interaction method with BEAM and SNPHarvester in artificial datasets simulating epistatic interactions and show that our method is more powerful to detect pairwise epistatic interactions than its competitors. We show results of the application of information interaction method to the WTCCC breast cancer dataset. Our results are validated using permutation tests. We were able to find 89 statistically significant pairwise interactions with a p-value lower than 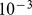. Even though many recent algorithms have been designed to find epistasis with low marginals, we observed that all (except one) of the SNPs involved in statistically significant interactions have moderate or high marginals. We also report that the interactions found in this work were not present in gene-gene interaction network STRING.

## Introduction

The availability of ever more extensive genetic information has spurred intense research on the search for the genetic factors that influence common complex traits. Genome Wide Association Studies (GWAS) aim at discovering associations between genetic factors and complex traits such as diseases. In GWAS, hundreds of thousands of Single Nucleotide Polymorphisms (SNPs) are analyzed to determine whether they are associated with the disease or conditions of interest. Due to limitations on the data, these analyses are usually performed using single SNP statistical tests and correcting for multiple testing.

This approach has severe limitations since epistatic interactions of SNPs are very important in determining susceptibility to complex diseases. Existing methods for SNP interaction discovery perform poorly when marginal effects of disease loci are weak or absent. As an example of a case where this may happen, it has been suggested that many genes with small effects rather than few genes with strong effects contribute to the development of asthma. The problem is that the individual effects of the interacting SNPs may be too small to be detected with the most commonly used statistical methods. Therefore, there is a need for more powerful methods that are able to identify interactions between SNPs with low marginal effects.

A number of different methods have been used to find epistatic interactions, including statistical methods (e.g. ATOM [1]), search methods (e.g. BEAM [2] or SnpHarvester [3]), regression methods (e.g. Lasso Penalized Logistic Regression [4]) and machine learning methods (e.g. [5], MegaSNPHunter [6] or decision tree based methods [7]). Classic methods such as Logistic Regression have been pointed as appropriate methods to consistently estimate the strength of association between a predictor and disease [8] [9]. An additional advantage of regression methods is the ability to deal with population structure by including principle components as covariates [10]. However it has also been stated that logistic regression has limited power for modelling high-order non-linear interactions that are likely important in the etiology of complex diseases [11]. Multivariate regression is not suited for problems with hundreds of thousands of variables. Intermediate strategies that permits fast computation while preserving the spirit of multivariate regression have been explored [4] [12]. The lasso penalty is an effective device for continuous model selection, especially in problems where the number of variables far exceeds the number of observations. The problem is that even these intermediate regression strategies are not prepared for dealing with the interactions between a large number of variables. That is why these methods are usually combined with filtering strategies such as selecting SNPs with high marginals and building the regression model considering only the interactions between these high-marginal SNPs [4]. However in this paper we chose not to use any filtering strategy in order not to loose any potentially important SNP on this step.

In this paper, we describe results obtained using a measure known as *information interaction*, that was used to identify pairs of SNPs that, together, provide a significantly higher risk of disease. Information interaction expresses the amount of information (reduction of entropy) provided by a set of variables, beyond that which is present in any subset of those variables. Using a computationally efficient approach, we compute the pair of SNPs with the highest information interaction, relatively to the phenotype of interest. To control for multiple hypothesis testing, the significance of the relevant results is validated using a permutation testing procedure. Information interaction has already been applied to epistasis detection [13] [14], however, those applications involved the use of a filtering step that may remove important SNPs. We show in our paper that it is possible to apply information interaction to the WTCCC breast cancer dataset without any filtering step.

The results obtained in artificial datasets show that the approach vastly outperforms existing methods such as SnpHarvester [3], and Beam [2]. Results obtained in the Wellcome Trust Breast Cancer dataset [15] have shown that the approach is applicable to the large amounts of data used in GWAS and that previously unknown, statistically significant, interactions can be discovered. We show also that the interactions that were statistically significant are composed of SNPs with moderate or large marginals.

## Materials and Methods

To test methods to find interactions with low marginals, we need to have a dataset that simulates these type of interactions. We selected simulated datasets that were tested with the SNPHarvester algorithm [3]. We used datasets in which there are multiple disease loci without marginal effects. Our argument is that if our method can detect these interactions in the artificial datasets, then it will be able to find similar types of interactions in real datasets such as the Wellcome Trust Breast Cancer Dataset.

### Artificial datasets

60 different epistatic models were the base for the generation of datasets in which disease loci do not have marginal effects. These epistatic models, firstly used in [16] use different parameter values for parameters such as heritability (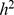) and minor allele frequency (MAF). Heritability ranges from 0.025 to 0.4 and MAF ranges from 0.2 to 0.4. 100 datasets were generated for each disease model, each one with 200 cases, 200 controls and 1000 SNPs. In these datasets there is only a pair of interacting SNPs in positions 1 and 10 from left to right.

The datasets in which there are multiple disease loci without marginal effects try to simulate the expectation that there might exist multiple SNP-SNP interactions in the association studies. Eight hybrid models were used for the generation of the datasets. Each hybrid model is a mixture of five pure epistatic models with the same heritability and MAF. For example if a hybrid model HM1 consists of models 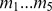, the first interaction is based on model 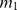, the second interaction based on model 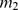 and so on. Thus there are five interactions in the HM that are simulated independently. 100 datasets were simulated for each hybrid model and each dataset contains 200 cases, 200 controls and 1000 SNPs. The 10 SNPs that belong to the 5 pairwise interactions are in positions (1,100), (201,300), (401,500), (601,700) and (801,900). Since these datasets with multiple loci are closer to what we may expect in reality, we will report the results of our methods in these datasets generated with hybrid models.

### Wellcome Trust Case Control Consortium breast cancer dataset

The artificial datasets are very important to test the methods and estimate their power. If we can develop a method that successfully detects the associations in the artificial datasets, then we can apply that method to real world datasets in order to detect associations with similar characteristics. The Wellcome Trust Case Control Consortium (WTCCC) breast cancer dataset is a large real world dataset with 1045 cases, 1476 controls and 15436 SNPs. We used only the SNPs that were also used in the WTCCC original publication [15] after data cleaning. Due to efficiency reasons it was also necessary to convert genotypes into binary variables. Each SNP is therefore coded with 2 bits which is enough since we use 3 values for the genotypes and 1 value for missing data. This encoding of the SNPs is not the same that was used in other works such as [13] or [14]. The reason for this is that the source code of the tool uses bitwise operations that make the execution to run much faster. As we will see in the results in the artificial datasets, our method, using this encoding, overcomes state-of-the-art methods BEAM and SNPHarvester in terms of power to detect pairwise epistatic associations.

### Classification methods

Our first approach was to select for study one artificial dataset that contains multiple disease loci, without marginal effects, and apply classification methods to see if it was possible to explain the phenotype based on the 1000 SNPs (10 of which are the disease loci).

We used WEKA to test several classification methods such as Alternating Decision Trees, Voted Perceptrons and Support Vector Machines. We also tested SMLR method (Sparse Multinomial Logistic Regression).

Alternating Decision (AD) Trees [17] are a generalization of the well known decision tree learning algorithm. The AD Tree learning algorithm is based on boosting. Boosting is a machine-learning meta-algorithm that tries to learn a strong classifier, based on weak classifiers. The models produced by AD Tree learning are relatively easy to interpret, especially if compared with other boosting procedures. AD Trees were originally proposed for binary classification, but posterior work extended it to work for multiple classes [18].

Voted perceptrons [19] are an evolution of the perceptron algorithm which is an Artificial Neural Network with only one neuron. Voted perceptrons store the list of all prediction vectors generated after each mistake. For each vector the method keeps the number of iterations it survives until the next mistake is made. This number is called the weight of the vector. To calculate a prediction, the binary prediction of each one of the vectors is computed and all predictions are combined by a weighted majority vote.

Support Vector Machines (SVMs) [20] are the basis of classification method based on creating a maximum-margin hyperplane in a transformed input space. The transformation of the input space is done implicitly by means of a kernel function. The parameters of the solution hyperplane are derived from a quadratic programming optimization problem using algorithms such as Sequential Minimal Optimization (SMO) [21].

Sparse Multinomial Logistic Regression (SMLR) [22] algorithm learns a multi-class classifier and simultaneously performs feature selection to identify a small subset of features relevant to the decision. The learned classifier reports the probability of a sample belonging to each of the 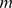 classes given 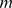 sets of feature weights, one for each class.

### Fleuret method

Fleuret published a fast binary feature selection technique (that we call the Fleuret method) based on Conditional Mutual Information Maximization (CMIM) [23] that we wanted to apply to our problem of detecting interacting SNPs with low marginals. This method is based on picking features that maximize their mutual information with the class to predict, conditional to any feature already picked. According to the author, this method selects features which are both individually informative and two-by-two weakly dependant.

The goal of this method is to select a small subset of features that carries as much information as possible. To measure the amount of information carried by the features, the Fleuret method uses Conditional Mutual Information. This information theory measure is based on another very important measure which is the Entropy 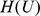 of a random variable, which quantifies the uncertainty of 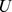. The conditional entropy 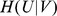 quantifies the remaining uncertainty of 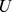, when 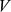 is known. If 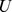 is a deterministic function of 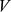 then 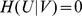. On the other hand if 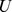 and 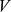 are independent, knowing 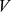 does not tell anything about 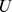 and 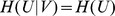. The Conditional Mutual Information is given by Equation 1:
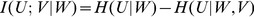
(1)


This value can be seen as the difference between the average remaining uncertainty of 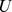 when 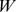 is known and the same uncertainty when both 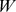 and 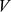 are known. If 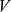 and 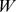 carry the same information about 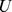, the conditional mutual information is zero. On the other hand if 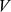 brings information about 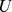 that is not already contained in 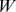, 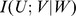 is different from zero.

The standard implementation of the algorithm is done by keeping a score vector 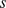 which contains for every feature 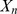, after the choice of feature 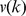, the score 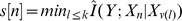. The score table is initialized with 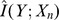 and the algorithm picks at each iteration the feature 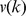 with the highest score. Every score 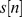 is then refreshed by taking the minimum of 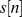 and 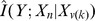. Fleuret proposed a faster implementation which is based on the fact that the score vector can only decrease when the process goes on and bad scores may not need to be refreshed. This faster implementation produces the same results as the standard implementation described.

### Information interaction pairwise search method

The application of a systematic search over all possible pairs of SNPs came as a natural idea after the bad performance of the Fleuret method. If we are not able to have any type of information to guide the choice of our first disease locus, that means we need to consider all the pairs of SNPs, to decide which ones may be interacting in a way that help us to explain the phenotype.

The choice of information interaction as the metric to evaluate if a pair of SNPs is associated with the phenotype arose as a natural option. We define that a pair of SNPs 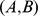 is associated with the phenotype with a user-defined threshold 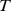 if and only if 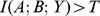. I is the information interaction (or synergy) and expresses the amount of information bound up in a set of variables, beyond that which is present in any subset of those variables (Equation 2).
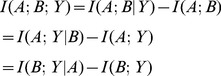
(2)


If the information that SNP 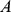 provides about class 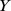 is higher if we know SNP 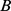 than it is if we do not know SNP 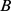, then this additional information is the interaction information or synergy between the two variables 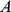 and 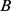 with respect to class 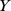.

In our experiments in Breast Cancer dataset, the user-defined threshold 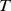 is defined by running 1000 permutation tests and selecting the highest interaction information value found. This way, all our selected interactions will have a p-value greater than 0.001.

### Mutual information search method

We also applied mutual information to all SNPs in order to find single SNP associations to the phenotype and be able to compare the results with information interaction method.

Similarly to the information interaction method, a SNP 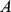 is associated with the phenotype with a user-defined threshold 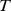 if and only if 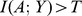. 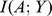 is the mutual information and expresses the amount of information that is shared between 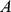 and 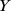 (Equation 3).
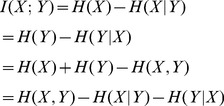
(3)


In our experiments in Breast Cancer dataset, the user-defined threshold 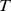 is defined by running 1000 permutation tests and selecting the highest mutual information value found. This way, all our selected single SNP associations will have a p-value greater than 0.001.

### Permutation testing

Permutation testing is a non-parametric procedure for determining statistical significance based on rearrangements of the labels of a dataset. It is a robust method but it can be computationally intensive. A test statistic, which is computed from the dataset, is compared with the distribution of permutation values. These permutation values are computed similarly to the test statistic, but under a random rearrangement of the labels of the dataset. Permutation tests can help reduce the multiple testing burden [24] and can be used to compare statistical tests [1].

In bioinformatics, permutation tests have become a widely used technique. The reason for this popularity has to do with its non-parametric nature, since in many bioinformatics applications there is no solid evidence or sufficient data to assume a particular model for the obtained measurements of the biological events under investigation [25].

## Results

### Application of classification methods to artificial datasets

The application of the classification methods to our selected artificial dataset did not produce good results. In fact, as we can see from Table 1 none of the methods achieved a 10-fold cross validation accuracy above 56.5%.

**Table 1:** 10-Fold Cross Validation Accuracies of the classifiers applied to the Artificial dataset.

AD Trees	55.5%
Voted Perceptron	54.5%
SVMs	51.5%
SMLR	56.5%

We also performed experiments using feature selection methods in WEKA. The 10-fold cross validation accuracies improved, but none of the variables selected by the feature selection methods corresponded to the disease loci. We tried several feature selection methods available on WEKA and none was able to identify some of the 10 disease loci.

**Figure 1:**
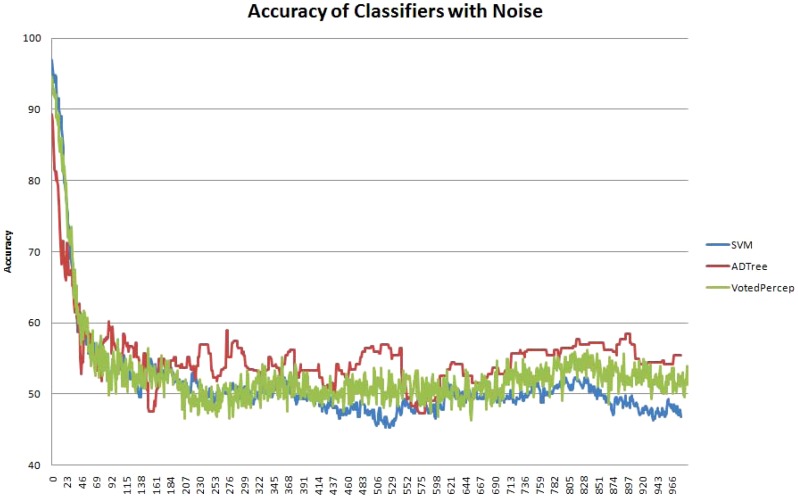
10-Fold Cross Validation Accuracy of classification methods with the addition of noisy variables.

A question that arised was how the performance of classifiers behaves when we add more noisy variables. To study that we performed a study in which we started by training a classifier with the 10 disease loci. We then add one noisy variable at each step and retrain the algorithm using the same training parameters. Even taking into account that the training parameters are not adjusted when we add more variables, it is interesting to see how the 10-fold cross validation accuracy rapidly degrades with the addition of noisy variables (see Figure 1).

We conclude that classification methods are not capable of identify the relevant factors when many irrelevant attributes are present.

### Application of fleuret method to artificial datasets

In our experiments, we used an implementation of the Fleuret Method available on the author's web page http://www.idiap.ch/~fleuret. This tool is able to perform feature selection with the Fleuret method and train/test a bayesian or a perceptron classifier. We were interested only in checking if the feature selection method was able to find the interacting SNPs of two artificial datasets, one with two interacting loci and the other with a total of 10 disease loci. However, the Fleuret method was not able to detect any of the interacting SNPs on these two datasets.

These bad results may happen because the Fleuret method initializes the score table 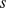 with mutual information 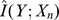. At the first step the algorithm greedily chooses the feature 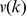 with the highest mutual information. This first step selects the wrong variable in SNPHarvester datasets, since the disease loci marginals are very low. This has an impact on the following steps since each disease locus does not add new information about the class to the wrongly selected SNP. As a consequence, the algorithm will continue to make wrong decisions.

The first steps are crucial and that is why we propose another approach that is based on testing all the possible pairs and evaluate them with Information Theory measures.

### Application of information interaction method to artificial datasets

**Figure 2:**
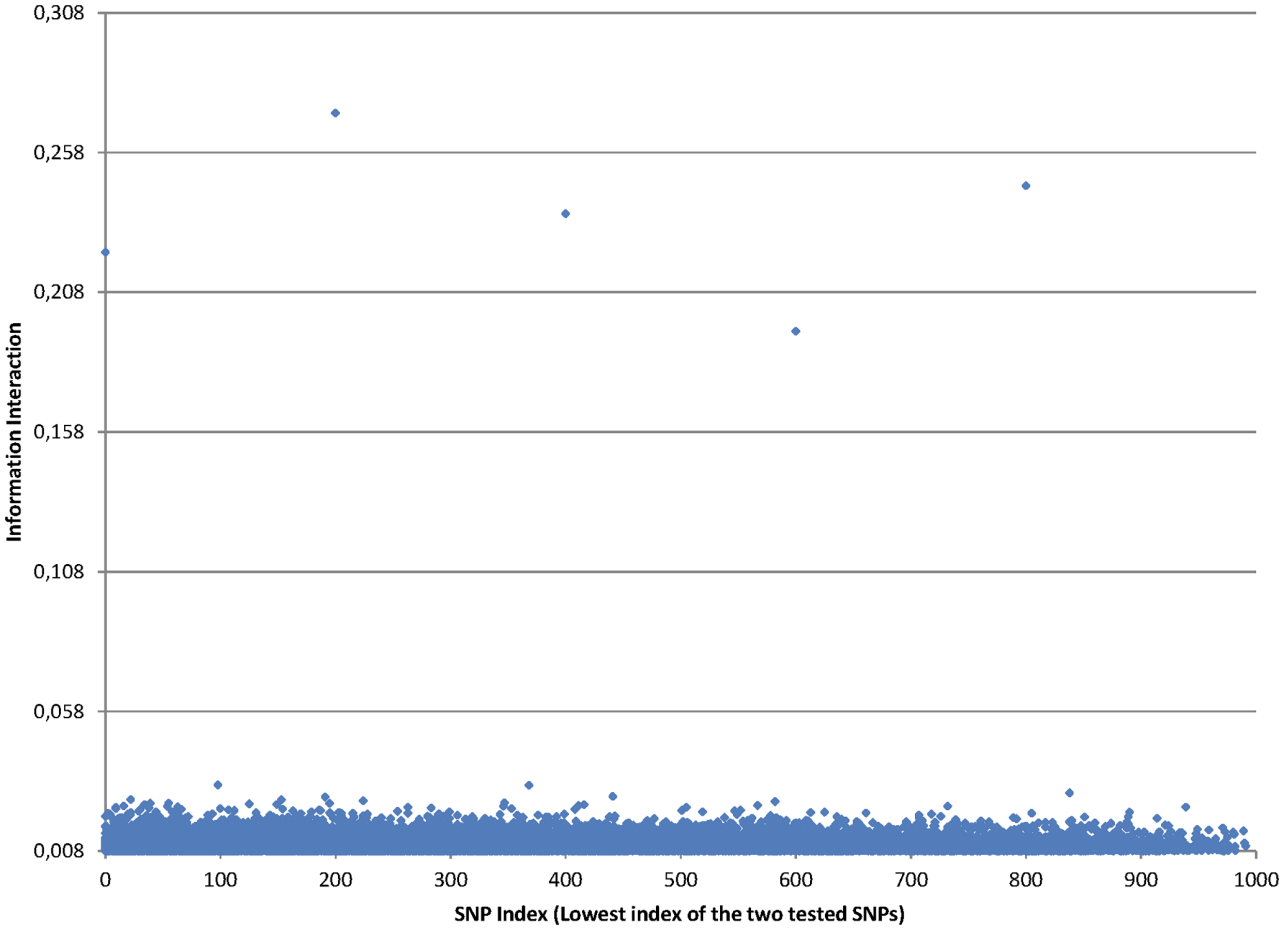
Information Interaction values for all pairs of SNPs 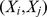 in artificial dataset. 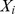
 is on the horizontal axis and Information Interaction is on the vertical axis. The 5 interacting pairs are very explicit, with information interaction values above 0.19.

**Figure 3:**
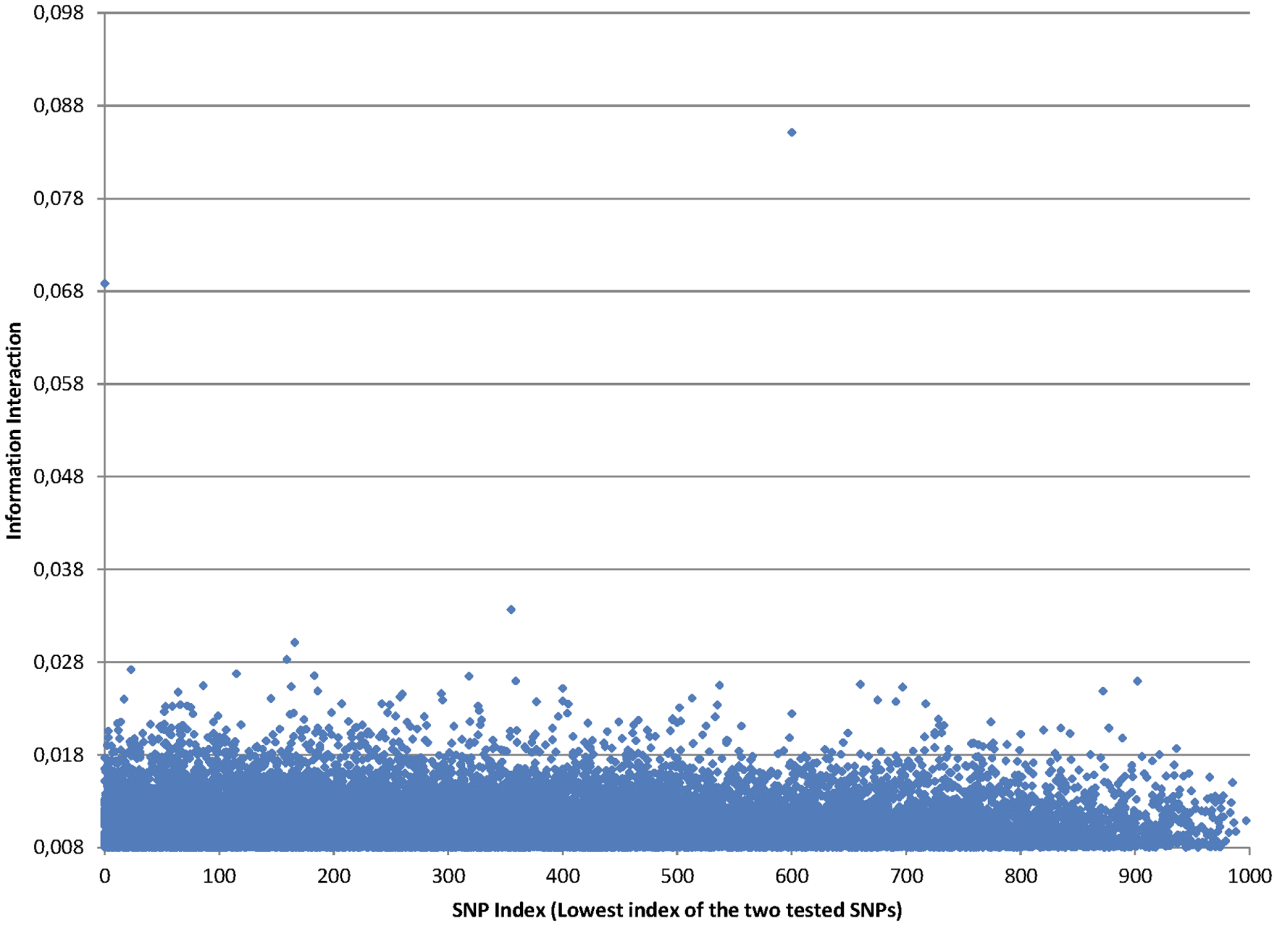
In this dataset, two pairwise interactions seem evident, but the other three are difficult to detect.

We applied information interaction metric to all possible pairs of SNPs in one artificial dataset and found that there was a big difference between values of true disease loci pairs and other noisy pairs of SNPs. It was possible to define a threshold that could perfectly distinguish between true disease loci pairs and not associated SNPs. Figure 2 shows the results obtained with Information Interaction method on one artificial dataset. The distinction between true disease loci and other SNP pairs is very explicit making it possible to detect the 5 pairwise interactions. We can see 5 points representing the 5 pairs of SNPs that are causing the disease in this simulated dataset. The minimum information interaction value of the 5 pairs of SNPs is 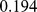 while the highest information interaction value of all the other pairs of SNPs is 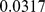. There are however other harder artificial datasets in which it is not so clear to detect all the 5 pairwise interactions as shown in Figure 3.

The results obtained in the artificial dataset, encouraged us to apply to all the datasets that were generated with equal or different simulation parameters. We could then compare the results obtained with the results of SNPHarvester and BEAM methods.

One of the main issues in applying information interaction (II) search is the choice of threshold 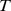. In preliminary experiments we used three variations. The first one was already described and is based on using a fixed value of 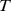 to all the datasets. However, in different datasets this value of 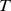 might not be the most appropriated. That is why se decided to use a permutation testing strategy to select 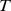. Our method is as follows: For each dataset we run 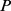 permutations and select the maximum II value between all the interactions. We multiply that maximum value by a given constant 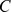 (we used 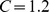 in our experiments) and that resulting value is the threshold 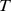 that we are going to use for that particular dataset. In our preliminary experiments we tested the number of permutations 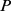 for threshold selection to be 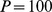 and 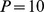. We decided to use 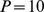 in our experiments.

**Figure 4:**
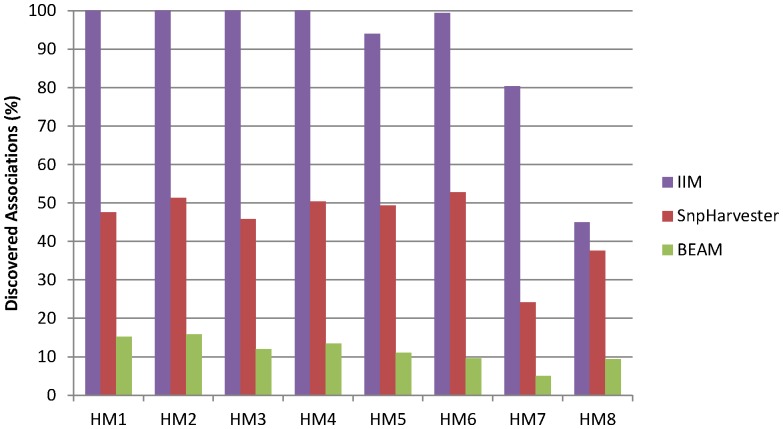
Comparison of Information Interaction Search with 10 permutations for threshold selection with SNP Harvester and BEAM in terms of Power.

We performed a comparison of our method with the results reported in [3] both for SNP Harvester and BEAM. In this comparison we used the Information Interaction Method (IIM) with a permutation strategy for threshold selection using 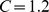 and 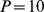. The results of the comparison are shown on Figure 4.

**Figure 5:**
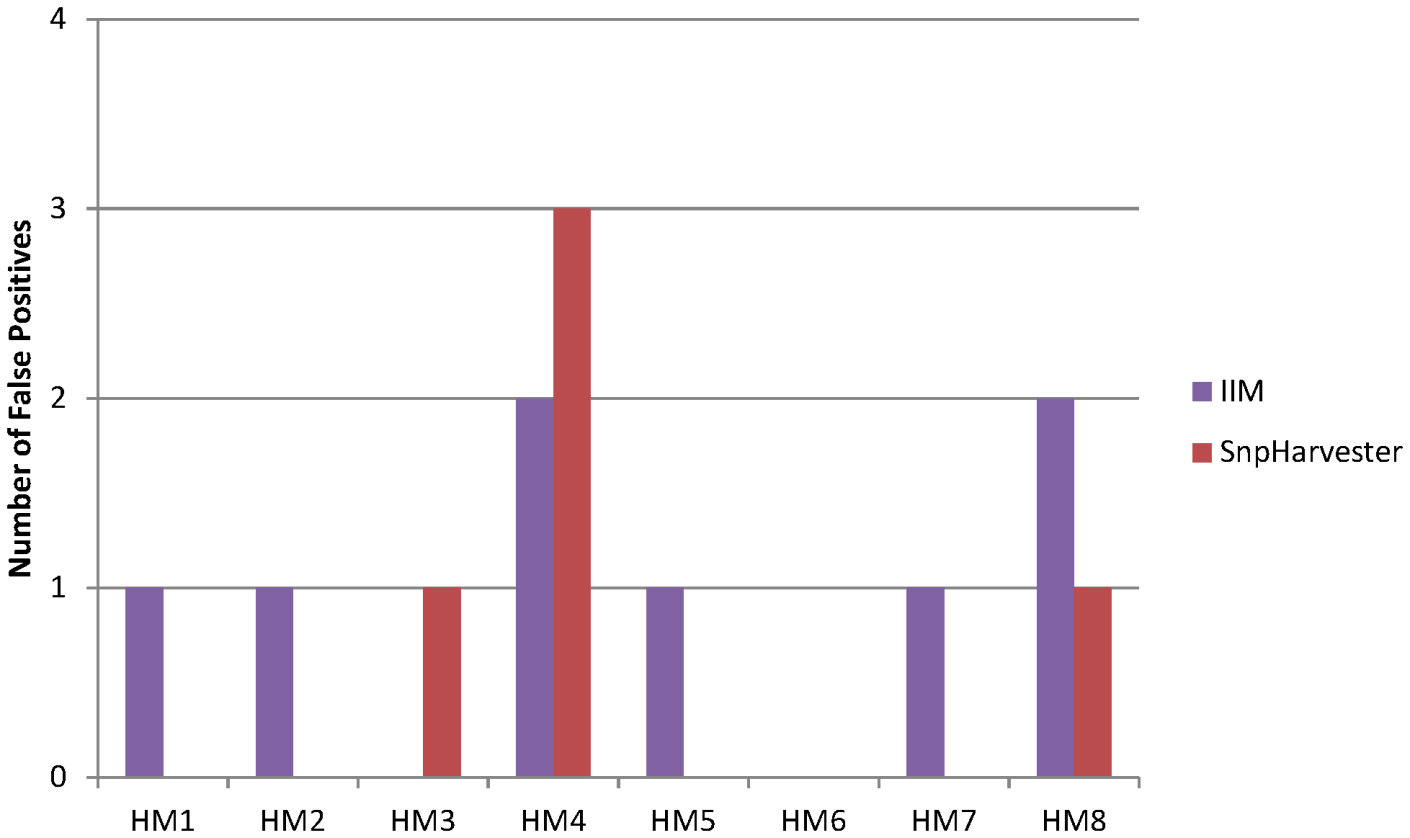
Comparison of II Method and SNP Harvester in terms of False Positives. SNP Harvester false positives were obtained by inspecting SNP Harvester results files that are available in http://bioinformatics.ust.hk/SNPHarvester.html.

As we can see, our Information Interaction method (IIM) outperforms both SNP Harvester and BEAM in terms of power. In Hybrid Model 8 our method has more problems in discovering the interactions because the signal is more dispersed in the noise. However, even in those harder conditions, our method performed better than SNP Harvester and BEAM. We also show in Figure 5 the comparison between our method and SNP Harvester in terms of the generation of false positives. As we can see, both methods generate few false positives. In a total of 800 datasets, SNP Harvester discovers a total of 5 false positives while our method discovers 8.

Information Interaction search showed to be a very powerful method to detect interactions between SNPs with low marginals and is therefore a very valid option to apply to real GWAS data.

### Application of regression methods to WTCCC breast cancer dataset

We tried to apply regression on the SPSS tool, but it was hardly possible: due to the high number of variables the software blocked (in a computer with 8 Gigabytes of RAM). We decided then to use statistical tool R in order to perform our experiments with regression and the same problem occurred given the high dimensionality of the parameters space. The methodology that we were able to run successfully was to use Bayesian Information Criterion (BIC) as the model selection criterion. When we learn the model of marginal effects using Lasso Penalized Logistic Regression and BIC, only 38 variables are selected. We then build the data matrix with all the interactions between these 38 selected variables and trained the model with Lasso and BIC. The final model includes 37 (of the 38) marginal variables and 11 interaction variables. This methodology has the disadvantage of eliminating SNPs with small effects that can be involved in interactions with low marginals.

### Application of information interaction method to WTCCC breast cancer dataset

**Figure 6:**
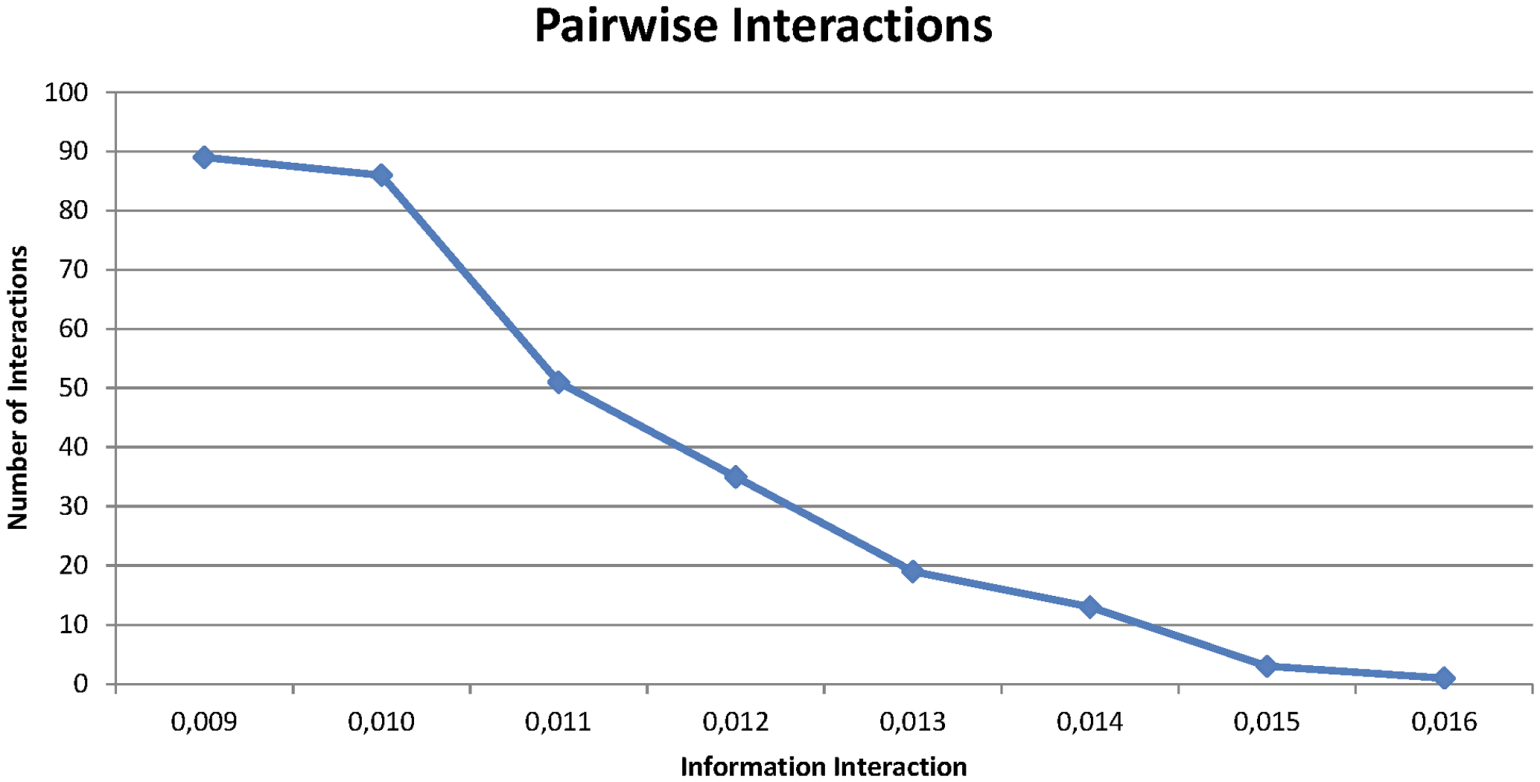
Accumulated number of pairwise interactions detected with Information Interaction in WTCCC Breast Cancer Dataset with 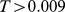 and 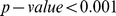.

After the pre-processing of the WTCCC Breast Cancer dataset, we applied the information interaction method. To determine the threshold 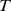 to use, we ran 1000 permutations tests. In each permutation test we applied the information interaction measure to all possible pairs of variables. At the end we selected the highest value 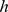 of information interaction between all pairs of SNPs discovered in the 1000 permutation tests. We then used a value of 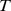 greater than 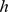 so that each interaction discovered is statistically significant at the 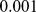 level of confidence. In our permutation procedure 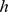 was found to be 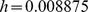. We then fixed 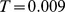. The distribution of information interaction values, greater than 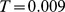, found in the original WTCCC Breast Cancer dataset is shown in Table 2 and Figure 6.

**Table 2:** Number of SNP pairs detected in the WTCCC Breast Cancer dataset with 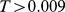.

Information Interaction	Number of SNP Pairs
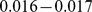	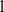
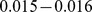	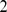
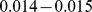	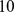
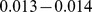	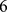
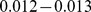	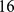
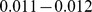	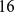
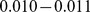	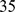
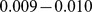	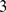

**Figure 7:**
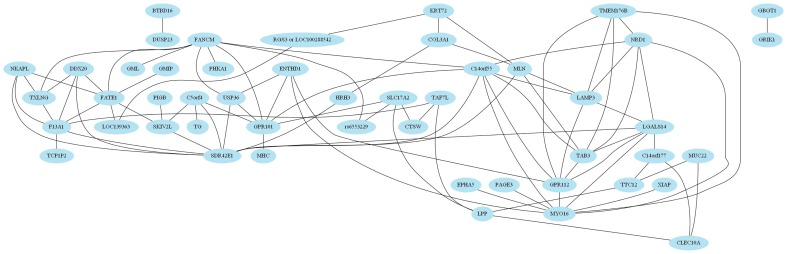
Graph representing the gene-gene interactions found with information interaction method on the WTCCC Breast Cancer dataset.

Figure 7 shows the network of interactions found with information interaction method in the WTCCC Breast Cancer dataset. Each SNP that was found with IIM is represented in the graph by a node that has the name of the gene to which the SNP belongs to. For each pairwise interaction discovered, we draw an edge between the nodes that correspond to the SNPs involved.

The null hypothesis that is considered in this work is that the phenotype is independent from the SNPs. If this is true, then we can make permutations of the phenotype and the distribution of information interaction values would be similar. However we observe that in our 1000 permutation tests it was not possible to find any II value above 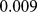. On the other hand we can see that in our original dataset there are 89 pairs of SNPs that have an II value higher than 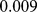. This means that we can use any of these 89 pairs of SNPs to reject the null hypothesis with a confidence of 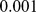, which is our p-value.

### Application of mutual information method to WTCCC breast cancer dataset

**Figure 8:**
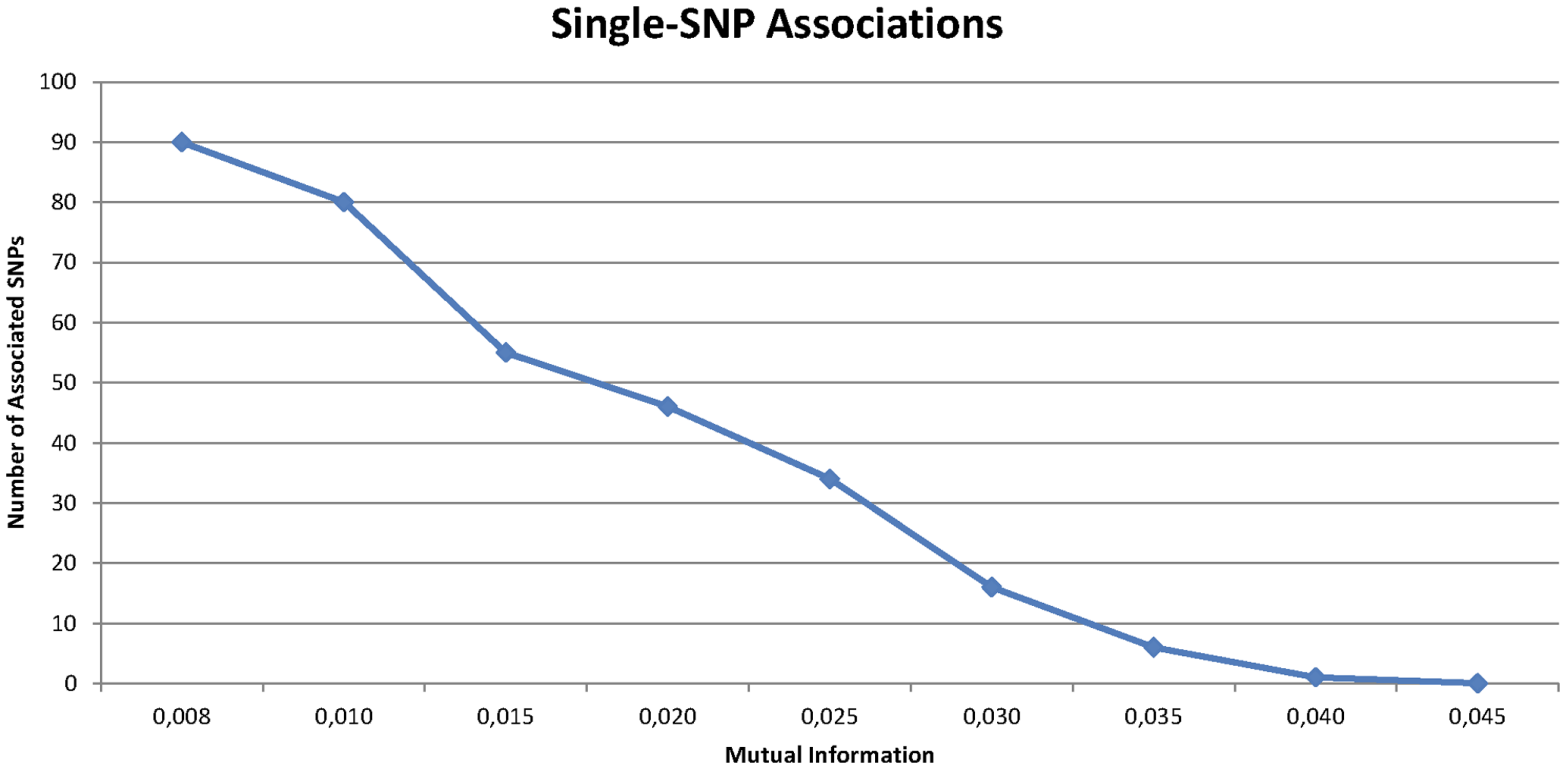
Accumulated number of single-SNP associations detected with Mutual Information in WTCCC Breast Cancer Dataset with 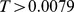 and 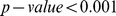.

We also applied the mutual information method to the WTCCC Breast Cancer dataset. As already mentioned, we used 1000 permutation tests to determine the threshold 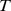. In each permutation test we applied the mutual information measure to each SNP and the phenotype. At the end we selected the highest value 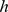 of mutual information between each SNP and the phenotype in all the 1000 permutation tests. We then used a value of 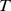 greater than 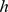 so that each association discovered is statistically significant at the 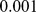 level of confidence. In our permutation procedure, 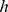 was found to be 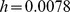. We then fixed 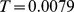. A total of 90 different SNPs were found to share information with the phenotype with a mutual information value above the fixed threshold 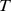. The distribution of mutual information values greater than 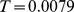, found in the original WTCCC Breast Cancer dataset is shown in Table 3 and Figure 8.

**Table 3:** Number of SNPs detected in the WTCCC Breast Cancer dataset with Mutual Information Method and a threshold 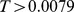.

Mutual Information	Number of SNPs
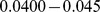	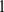
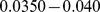	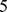
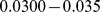	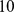
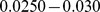	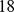
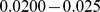	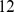
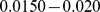	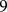
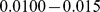	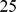
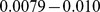	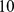

### Performance characteristics of information interaction method

The execution of the Information Interaction Method on the Wellcome Trust Breast Cancer dataset takes around 45 minutes in a 2.3 GHz AMD Opteron Processor. The execution of 1000 permutations in a single processor would take about a month, or 3 days if we use 10 processors. We also made some experiments with an Alzheimer dataset from the Alzheimers Disease Neuroimaging Initiative (ADNI) with 600000 SNPs and the execution after data quality procedures took around four days. We did not complete the execution of the 1000 permutation tests because we could conclude in advance that the results would not be statistically significant. The complete set of 1000 permutation tests would take several years to complete in a single processor, although it could be parallelized. Since most of the time it is not practical to have access to a large number of processors, there is still the need for methods that need less permutation tests in order to measure statistical significance. We are currently working on methods that obtain the estimates of the p-values with a much smaller number of permutation tests.

## Discussion

In our work, we adapted the source code from the Fleuret method in order to calculate information interaction over all possible pairs of SNPs. With this approach we benefited from the efficient calculations of conditional mutual information that was already developed. Even though computational efficiency is an important issue, because we want results in feasible time, it was not our major point of interest. We gave priority on designing a method that could give us some guarantees of finding pairwise interactions, in cases where both variables have low marginals, if they exist, even if we have to wait for the results. We saw from the results of the application of our method to the artificial datasets that our method was powerful when compared to other state of the art methods. Therefore, if that type of interactions exist in the real breast cancer dataset, our method would detect them. Another advantage of our information interaction method over stochastic methods is the fact that it is deterministic. One of the problems of stochastic methods is that they can give different results in different runs. Our method makes an exhaustive analysis of pairs of SNPs and gives always the same results.

In the artificial datasets that were used with the information interaction method, all the interactions had low marginals. Each SNP of the pairwise interactions did not have any statistically significant association with the phenotype when considered alone. We showed in our results that our method based on information interaction could find these pairs of SNPs more often than other state of the art methods such as SNPHarvester or BEAM.

The application of the mutual information method to the WTCCC breast cancer dataset found that there were 90 SNPs that individually shared information with the phenotype with a p-value 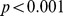. In addition, as was already reported, the application of the information interaction method to the WTCCC breast cancer dataset found 89 pairs of SNPs that, when considered together, share more information with the phenotype than when considered individually. A total of 49 different SNPs were involved in the 89 interactions. 48 of these 49 SNPs found with information interaction were also found with the mutual information method. The only exception was SNP rs660895 from MHC gene that was found with the information interaction method but not with the mutual information method. Table 4 summarizes the SNPs that were found with mutual information and information interaction methods.

**Table 4:** SNPs and their respective genes discovered with both IIM and MIM, only with IIM and only with MIM. Between parenthesis we have the number of SNPs in the respective category.

IM and MIM (48)		IIM only (1)		MIM only (42)	
SNP	Gene	SNP	Gene	SNP	Gene
rs1048347	BTBD16	rs660895	MHC	NCBI35_X_15303842	
rs1129923	DUSP23			rs10456324	LRRC16A
rs12003	GMIP			rs1059655	HLA-E
rs12665700	MUC22			rs1132200	TMEM39A
rs12833456	KRT72			rs1151687	OR2G2
rs1367580	FANCM			rs11558709	ENSG00000188699
rs1635	NKAPL			rs12984558	DHX34
rs17256042	TMEM176B			rs1385698	EDA2R
rs17319801	ENTHD1			rs1727	IFIT2
rs17641488	C5orf4			rs17280682	NLRP14
rs1800255	COL3A1			rs176024	MAGEC3
rs180223	TG			rs1788799	NPC1
rs197414	DDX20			rs1800280	DMD
rs2071299	SLC17A2			rs1800309	GAA
rs2073924	GBGT1			rs1804027	SP110
rs2273198	NRD1			rs1968956	ELTD1
rs2281820	MLN			rs2071307	ELN
rs2290344	PIGB			rs2072994	PTCHD2
rs2293877	C14orf55			rs2093066	BPIFB3
rs3088040	USP36			rs2207337	
rs363504	GRIK1			rs2227289	CD320
rs3764795	GML			rs2229995	APC
rs3787429	HRH3			rs2283432	FANCI
rs3810715	FATE1			rs2706762	PCYOX1
rs4826381	PAGE3			rs3012075	CTBP2
rs482912	LAMP3			rs3115572	
rs4830	LGALS14			rs3810510	JPH2
rs4905757	C14orf177			rs393414	
rs592229	SKIV2L			rs4826957	
rs5927629	TAB3			rs4827331	SYTL5
rs5930931	GPR112			rs4897783	FLJ46300
rs5931046	GPR101			rs5924658	PASD1
rs5951328	TAF7L			rs5930932	GPR112
rs5956583	XIAP			rs5951332	ARMCX4
rs5969783	TXLNG			rs595413	FAM217A
rs5983	F13A1			rs5955762	MAP3K15
rs598318	TCP1P2			rs599176	
rs604630	CTSW			rs631357	KIF17
rs6553229				rs652438	MMP12
rs6564956	SDR42E1			rs6525447	SLC7A3
rs664850	RGS3 or LOC100288542			rs662204	MHC
rs7054230	PHKA1			rs706107	SPINK4
rs723077	TTC12				
rs7349683	EPHA5				
rs7645635	LPP				
rs7879053	LOC139363				
rs90951	CLEC10A				
rs911973	MYO16				

Even though the scientific literature refers the need for methods to discover low marginal interactions, our results suggest that most epistatic interactions with relevance to breast cancer have moderate or high marginals. In addition none of the interactions discovered with the information interaction method were reported in the STRING network. We believe that this method should be applied to more breast cancer datasets and also to other datasets from other diseases in order to find more about the kind of epistatic interactions that exist in real diseases.
